# Evaluation of *Wuchereria bancrofti* GST as a Vaccine Candidate for Lymphatic Filariasis

**DOI:** 10.1371/journal.pntd.0000457

**Published:** 2009-06-09

**Authors:** Anandharaman Veerapathran, Gajalakshmi Dakshinamoorthy, Munirathinam Gnanasekar, Maryada Venkata Rami Reddy, Ramaswamy Kalyanasundaram

**Affiliations:** 1 Department of Biomedical Sciences, University of Illinois College of Medicine at Rockford, Rockford, Illinois, United States of America; 2 Department of Biochemistry, Mahatma Gandhi Institute of Medical Sciences, Sevagram, Maharashtra, India; McGill University, Canada

## Abstract

**Background:**

Lymphatic filarial parasites survive within the lymphatic vessels for years despite the complex immune environment surrounding them. Parasites possibly accomplish this by adopting various immunomodulatory strategies, which include release of glutathione-S-transferases (GSTs) that counteract the oxidative free radicals produced by the host. Since GSTs produced by parasites appear to be critical for the survival of parasites in the host, several studies evaluated the potential of parasite GSTs as vaccine candidates especially against schistosomiasis, fascioliasis and *Seteria cervi*. However, vaccine potential of GSTs of lymphatic filarial parasites has not been evaluated before.

**Methods/Principal Findings:**

In the present study, the *GST* gene was cloned from the third stage larval (L3) cDNA libraries of *Wuchereria bancrofti*, and recombinant GST (WbGST) was expressed and purified. Serum samples from individuals living in an endemic area were analyzed for their reactivity with rWbGST. These findings showed that sera from endemic normal individuals (EN) carry significant levels of anti-WbGST IgG antibodies compared to subjects who are microfilaraemic (Mf) or show symptoms of clinical pathology (CP). Isotype analysis of the anti-WbGST IgG antibodies showed a predominance of IgG1 and IgG3 antibodies in EN individuals. Subsequent functional analysis of the rWbGST showed that the rWbGST protein retained the enzymatic activity of GST and the antibodies in EN sera could inhibit this enzymatic activity. Similar results were obtained when anti-rWbGST antibodies raised in mice were used in the neutralization assay. *Brugia malayi* GST and WbGST show significant sequence similarity. Therefore, to evaluate the vaccine potential of rWbGST, we used *B. malayi* L3 as challenge parasites. Vaccine potential of rWbGST was initially evaluated by confirming the role of human and mice WbGST antibodies in an antibody dependent cellular cytotoxicity (ADCC) assay. Subsequent vaccination studies in a jird model showed that approximately 61% protection could be achieved against a *B. malayi* L3 challenge infection in jirds immunized with rWbGST.

**Conclusions:**

Results of this study show that rWbGST is a potential vaccine candidate against lymphatic filariasis. Nearly 61% protection can be achieved against a *B. malayi* challenge infection in a jird model. The study also showed that the WbGST protein retained the enzymatic activity of GST and this enzymatic activity appears to be critical for the survival of the parasite in the host.

## Author Summary

Lymphatic parasites survive for years in a complex immune environment by adopting various strategies of immune modulation, which includes counteracting the oxidative free radical damage caused by the host. We now know that the filarial parasites secrete antioxidant enzymes. Among these, the glutathione-S-transferases (GSTs) have the potent ability to effectively neutralize cytotoxic products arising from reactive oxygen species (ROS) that attack cell membranes. Thus, GSTs have the potential to protect the parasite against host oxidative stress. GSTs of several helminthes, including schistosomes, fasciola and the filarial parasite *Seteria cervi*, are also involved in inducing protective immunity in the host. The schistosome 28 kDa GST has been successfully developed into a vaccine and is currently in Phase II clinical trials. Thus, GST appears to be a potential target for vaccine development. Therefore, in the present study, we cloned *W. bancrofti* GST, and expressed and purified the recombinant protein. Immunization and challenge experiments showed that 61% of protection could be achieved against *B. malayi* infections in a jird model. *In vitro* studies confirm that the anti-WbGST antibodies participate in the killing of *B. malayi* L3 through an ADCC mechanism and enzymatic activity of WbGST appears to be critical for this larvicidal function.

## Introduction

Lymphatic filariasis is a mosquito borne infection caused by *Wuchereria bancrofti*, *Brugia malayi* or *Brugia timori* that affects 120 million people in 73 countries and another 1100 million people are at risk [1],[2]. Because of the gruesome pathology associated with this infection, lymphatic filariasis is considered as a major obstacle to economic development in endemic countries and identified as the second leading cause of permanent and long-term disability. Although excellent anti-filarial drugs are available, several rounds of mass treatment are necessary to reduce the levels of infection below those necessary to sustain transmission [3]. Therefore, additional preventive measures such as vector control and vaccine development are crucial to control the infection in endemic regions. A certain population of individuals (called endemic normal or EN) in the endemic area is refractory to the infection. These individuals carry high levels of antibodies against the parasite antigens which are believed to be protective [4]. Therefore, most of the vaccine studies were focused on identifying the molecules recognized by these antibodies.

Especially, antigens of the infective third stage larvae of filarial parasites are of special interest since they represent the first larval stage that enters into the human host. Thus, anti-parasitic mechanisms against these infective larvae can potentially prevent the infection. Previous studies show that both antibodies and effector cells are important in this anti-parasitic mechanism functioning via an antibody dependent cellular cytotoxicity (ADCC) mechanism [5],[6]. Studies have also demonstrated a role for antibody and/or complement in the *in vitro* and *in vivo* cytotoxic response to the larvae [7],[8].

Lymphatic filarial parasites reside inside the lymphatic system and bathe in lymph that carry immune cells and molecules, yet they survive for years without any major harm and appear to be not damaged by the oxidative free radicals released from the host cells. This is largely possible because of the ability of the parasite to produce and secrete molecules such as glutathione-S-transferases (GSTs), SOD, catalase, glutathione peroxidase, peroxiredoxins [9] that can neutralize cytotoxic products arising from reactive oxygen species (ROS) that attack on cell membranes.

Neutralizing the effect of these molecules by immunization or vaccination could affect the ability of the parasite to survive in the host. A protective role for immunization with GSTs from several helminth parasites including schistosomes, fasciola [10] and the filarial parasite *Seteria cervi* have already been established [11]–[13]. The schistosome 28 GST has been successfully developed into a vaccine against *S. mansoni* [14] and *S. bovis* [15],[16]. The mechanisms underlying the protection conferred following immunization with *S. mansoni* 28 GST appears to be due to an inactivation of the GST enzymatic activity [17]. Thus, neutralizing the activity of GST may be a strategy to induce protection against certain parasitic infections. Immunization of mice with *Fasciola gigantica* GST26 could confer from 77–84% protection against challenge infection in mice [18]. *F. gigantica* GST26 and *S. mansoni* GST28 show significant sequence similarity such that immunization of mice with recombinant *F. gigantica* GST26 could confer cross protection against challenge infections with *S. mansoni* in mice. Thus, GST appears to be a critical protein for the survival of the parasite in the host. Based on homology sequences Nathan *et al* [19] constructed the 3D structure of *W. bancrofti* GST (WbGST) and predicted sites for docking of GST inhibitors as a new approach to develop drugs against *W, bancrofti*. These studies suggest that WbGST may be a promising target for vaccine development. Therefore, in this study we have attempted to evaluate the vaccine potential of filarial GST.

## Methods

### *Brugia malayi* parasites

*B. malayi* parasites (L3, mf, adult male and adult female) used in this study were obtained from NIH/NIAID Filariasis reagent repository center, College of Veterinary Medicine, University of Georgia, Athens, GA.

### Human sera samples

Blood samples were collected from the following clinical groups after obtaining informed consent: 1) Endemic normal (EN) subjects, who are asymptomatic, non-microfilaraemic and reside in villages surrounding Sevagram, Wardha, India with no symptoms of the disease; 2) Symptom-free microfilaraemic carriers (MF) (identified positive by microscopic examination of night blood smear); 3) chronic pathology (CP) patients who are visiting the out-patient department of Kasturba Hospital and were showing clinical symptoms of filariasis. Non-endemic normal (NEN) blood samples were collected from undergraduate medical students coming from non-endemic areas (such as Himachal Pradesh and Jammu & Kashmir States of India) with no circulating parasites and no evidence of filarial disease who were registered at the time of their admission to Mahatma Gandhi Institute of Medical Sciences, Sevagram. Use of human subjects in this study was approved by the IRB committees at Department of Biochemistry, Mahatma Gandhi Institute of Medical Sciences, Sevagram, India and at the University of Illinois, Rockford, USA. Sera separated from the blood samples were stored at −20°C freezer until use.

### Construction of pRSET A-*W. bancrofti* GST (WbGST) expression plasmid

*WbGST* gene was amplified from WbL3 cDNA library using insert specific primers (Forward: 5′CGCGGATCCATGAGTTATAAACTGAC3′ and Reverse: 5′CCGGAATTCTCACTGTTTTCCATTTCC3′). PCR parameters were as follows: 94°C of denaturation for 30 s, 50°C of primer annealing for 30 s, 72°C of primer extension for 30 s, and cycled for 30 cycles; a final extension of 5 min was performed at 72°C. PCR products obtained were digested with *Bam* HI and *Eco* RI enzymes and ligated to similarly digested T7 expression vector pRSET A (Invitrogen, Carlsbad, CA). Insert DNA was sequenced on both strands to ensure authenticity of the cloned nucleotide sequence. The pRSET A-*WbGST* construct was transformed into *E. coli* BL21 pLysS to express the recombinant protein.

### Expression and purification of rWbGST

The *E. coli* BL21 containing pRSET A-*WbGST* construct was grown to A_600_ ∼0.6 in LB medium containing ampicillin/chloramphenicol at 37°C and the expression of rWbGST was induced by the addition of isopropyl-β-D-thiogalactopyranoside (IPTG) to a final concentration of 1 mM IPTG. The culture was further incubated for another 3 h at 37°C. Bacteria were pelleted by centrifugation and resuspended in phosphate-buffered saline (PBS; 150 mM NaCl, 50 mM Na_2_HPO4, 50 mM NaH_2_PO4 pH 8.0). Finally, the cells were lysed by sonication and the debris was precipitated by centrifugation at 12000 rpm at 4°C for 20 min.

Collected supernatant was passed through immobilized cobalt metal affinity column chromatography (Clontech, Mountain View, CA) to purify the His tagged rWbGST as per manufacturer's instructions. The expression pattern and purity were analyzed by 15% SDS PAGE. Presence of Histidine-tag in the purified protein was detected using penta His-HRP monoclonal antibodies (Qiagen, Valencia, CA). Concentration of protein was estimated by micro BCA method (Thermo Fisher, Rockford, IL).

### Determination of GST enzyme activity

GST(s) enzyme activity was determined spectrophotometrically at 340 nm by measuring enzymatic conjugation of glutathione with 1-chloro-2, 4-dinitrobenzene (CDNB) [20]. Nonenzymatic conjugation with addition of reaction mixtures without the enzyme served as controls. Under standard assay conditions, the reaction mixture contained 100 mM phosphate, pH 6.5, 1.0 mM CDNB in ethanol, 1.0 mM GSH, and enzyme protein. A unit of enzyme activity was expressed as the amount that catalyzes the formation of 1 µmol S-2, 4-dinitrophenyl-GSH adduct per minute, using a molar extinction coefficient of 9.6 mM^−1^ cm^−1^ for CDNB.

### Screening human sera for WbGST antibodies

Sera from different groups of subjects from the endemic and non-endemic regions were screened for the presence of antibodies against recombinant WbGST using both Immunoblot analysis and an indirect ELISA. For immunoblot analysis, rWbGST protein was separated on 15% SDS-PAGE, transferred to nitrocellulose membrane and blocked overnight with 5% skimmed milk. After washing, the membrane strips were incubated individually with diluted (1∶50) pooled sera from MF, CP, EN or NEN individuals. The bound antibodies were probed with HRP labeled mouse anti-human IgG and color was developed with DAB/H_2_O_2_ chromogenic-substrate complex (Thermo fisher Scientific).

An indirect ELISA was performed to measure the level of IgG antibodies in individual sera sample. A total of 176 sera samples belonging to 45 microfilaraemics (Mf), 45 chronic pathology (CP), 43 endemic normal (EN) and 39 non endemic normal (NEN) individuals were screened for anti WbGST IgG antibodies using rWbGST as antigen in the indirect ELISA. Briefly, wells of an ELISA plates were coated with 1 µg/ml of rWbGST. After blocking the non-specific binding sites human sera samples were added at 1∶100 dilutions. After washing the plates, horse radish peroxidase labeled anti human IgG (1∶10000; Sigma, St. Louis, Missouri) was added and color was developed using OPD substrate.

Isotype of anti-rWbGST IgG antibodies were also analyzed in the sera of various study groups by indirect ELISA using biotinylated mouse monoclonal antihuman IgG1, IgG2, IgG3 and IgG4 and probed with avidin-horse-radish-peroxidase (Sigma, St. Louis, Missouri) as the secondary antibodies.

### Immunization of mice and jirds with rWbGST

Use of animals in this study was approved by the IACAUC committee at the UIC College of Medicine, Rockford and by the Institutional Animal Ethics Committee (IAEC) under the guidelines of Committee for the purpose of control and supervision on experiments on Animals (CPCSEA), Wardha, India. Five 2 months old male Balb/c mice were immunized intraperitoneally for antibody generation and seven two-months-old jirds were immunized intraperitoneally with 15 µg of rWbGST per animal in 100 µl of 0.5 M PBS along with equal volume of alum (Thermo fisher Biotech) as adjuvant. Three doses of the antigen were given at 15 days interval followed by one booster dose. Age matched control mice and jirds (five animals per group) received alum alone. One week after the final booster dose, sera were collected and tested for anti-WbGST IgG antibody levels. These sera samples were also used in downstream ADCC assays to determine their ability to kill *B. malayi* L3s *in vitro*.

### Analysis of antibody levels

Total anti WbGST IgG antibody levels in the sera samples of mice and jirds were determined using an indirect ELISA as described above. HRP-labeled goat anti-mouse IgG (ThermoFisher Scientific) was used as the secondary antibody. In addition, we also determined the isotype of anti-WbGST IgG antibodies (IgG1, IgG2a, IgG2b and IgG3) in the mice sera using a kit purchased from ThermoFisher Scientific. Color was developed using ABTS (2,2′-azinobis,3-ethylbenzothiazoline-6-sulfonic acid) substrate and absorbance was measured at 405 nm in a microplate reader.

### Expression of *GST* in different life cycle stages of *W. bancrofti*

*WbGST* gene was PCR amplified from the cDNA libraries of third stage infective larvae (L3), microfilariae (MF) and adult female (AF) of *W. bancrofti* using gene specific forward and reverse primers. PCR conditions used were an initial denaturation at 94°C for 5 min, 30 cycles (30 s at 94°C, 30 s at 50°C and 30 s at 72°C) of amplification, and a final extension at 72°C for 5 min. The amplified products were analyzed on a 1% agarose gel.

### Depletion of anti WbGST antibodies from sera

Anti-WbGST antibodies in the sera of immunized mice and in the pooled sera of EN individuals were depleted by passing the sera over rWbGST coupled to Cobalt IMAC resin (Clontech). Briefly, 1 mg of his-tagged rWbGST was coupled to the resin, washed and incubated overnight at 4°C with about 200 µl of neat sera. Supernatant was collected by centrifuging the resin mixture for 2 min at 750 rpm. Depletion of anti-WbGST antibodies were confirmed using an ELISA as described above.

### Inhibition of enzymatic activity

Evaluation of the inhibition of GST catalytic activity by anti-WbGST antibodies was determined as described previously using chloro-2, 4-dinitrobenzene as the substrate for GST activity [21]. For inhibition assay, 6.5 µg of rWbGST suspended in 25 µl was incubated with 20 µl of sera samples for 1 h at 37°C followed by four h incubation at 4°C. Sera samples used in this assay were (i) pooled sera samples from EN individuals, (ii) sera from immunized mice, (iii) WbGST antibody depleted sera from immunized mice and (iv) WbGST antibody depleted pooled sera from EN individuals. After incubation, the residual enzymatic activity was determined as described above. Results were compared with control samples (preimmune mice sera or NEN sera) that were processed similarly.

### Neutralization of GST activity with cDNB

For neutralization of GST activity in the parasite, we followed the method described by [22]. Briefly, *B. malayi* L3s were incubated with 14 µM of 1-chloro-2, 4-dinitrobenzene for 2 hrs at 37°C. Following these the cDNB-treated L3s were used in antibody dependent cell cytotoxicity.

### *In vitro* antibody-dependent cellular cytotoxicity (ADCC) assay

To determine the cytotoxic effects of anti rWbGST antibodies against *B. malayi* L3s, we performed an *in vitro* ADCC assay as described previously [23]. Sera samples used in this assay were from (i) EN individuals, (ii) immunized mice, (iii) WbGST antibody depleted sera from immunized mice and (iv) WbGST antibody depleted sera from EN individuals. Pre-immune or NEN sera samples served as negative controls. Briefly the ADCC assay was performed by adding 8–13 L3 of *B. malayi* to a suspension of 2×10^5^ peritoneal exudates cells (PEC) collected from normal Balb/c mice or PBMC's collected from NEN (WbGST antibody negative) individuals. Approximately, 50 µl of the sera samples were diluted with 50 µl of RPMI 1640 media and added to the suspension to make a final volume of 200 µl. The suspension was then added to wells of a 96 well culture plates (ThermoFisher) and incubated for 48 h at 37°C and 5% CO_2_. Larval viability was determined under a light microscope 48 h after incubation. Parasites those were limpid and straight with no movements were counted as non-viable and if they were still limpid and straight for the next 8 hours at 37°C, they were counted as dead. The live larvae were still active. We followed the method used by [24]. Results were expressed as the ratio of immobile or dead parasites to the total number of parasites recovered within each experiment. In some studies we also used cDNB-treated *B. malayi* L3s to neutralize the GST activity in the parasite and measure ADCC.

### Heterologous challenge experiments in jirds

rWbGST immunized and control groups of jirds were challenged intraperitoneally with 100 *B. malayi* L3 per animal10 days after the last dose of immunization. We decided on the heterologous challenge for three reasons (1) sequence similarity of WbGST and BmGST (2) lack of an animal model for *W. bancrofti* and (3) due to the fact that *W. bancrofti* infections are more prevalent in endemic countries than *B. malayi*.. 120 days after challenge infection, animals were euthanized by intraperitoneal injection of sodium pentabarbitol. Thorax and abdomen were opened to collect adult filarial worms from abdominal cavity, testes, heart, lungs and other organs. Number of adults worms recovered from each animal was recorded.

### Statistical analysis

Statistical analysis was performed using XL STAT software v.7.5.2 (Kovach Computing Services, Anglesey, UK). Statistical significance between comparable groups was estimated using appropriate non-parametric tests, with the level of significance set at p<0.05.

## Results

### Expression and enzymatic activity of rWbGST

**Figure 1:**
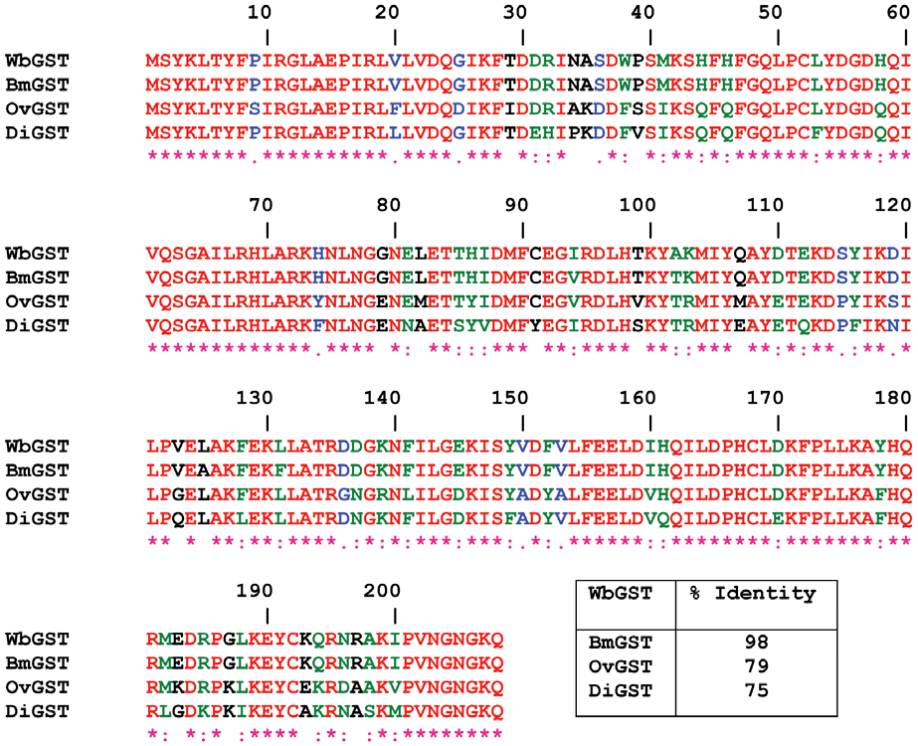
Multiple sequence alignment of WbGST. Multiple sequence alignments (CLUSTAL) of the amino acid sequences of GST family of proteins from *W. bancrofti* (WbGST; accession no. AY195867), *Brugia malayi* (BmGST, accession no. Y12788), *Onchocerca volvulus* (OvGST, accession no. L28771) and *Dirofilaria immitis* (DiGST, accession no. P46426). The amino acid positions are numbered above the amino acid sequences. Multiple alignment results show that GSTs from filarial parasites are highly identical to each other. (*) red color denotes identical amino acids, (:) green color denotes strongly similar amino acids and (.) blue color denotes weakly similar amino acids.

**Figure 2:**
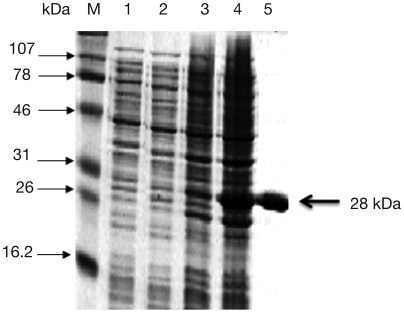
Expression and purification of rWbGST. Cultures of *E. coli* containing pRSETA and rWbGST expression construct were induced with 1 mM IPTG. Following induction, rWbGST was purified from the cultures using a cobalt metal affinity chromatography column. Approximately 1 µg of the purified protein was then separated in a 15% SDS-PAGE and stained with coomassie brilliant blue R250. Lane 1- pRSET-A uninduced, Lane 2- pRSET-A induced, Lane 3- rWbGST uninduced, Lane 4- rWbGST induced, Lane 5- Purified rWbGST and Lane M is protein molecular weight marker.

Multiple sequence alignments of the amino acid sequences of GST family of proteins from *W. bancrofti* (WbGST; accession no. AY195867) were compared with other filarial parasites such as *B. malayi* (BmGST, accession no. Y12788), *Onchocerca volvulus* (OvGST, accession no. L28771) and *Dirofilaria immitis* (DiGST, accession no. P46426). These analyses showed significant sequence identity between the various GSTs (Figure 1). Amino acid sequence analysis showed that WbGST shares 98% identity with *B. malayi* GST, 79% identity with *O. volvulus* GST and 75% identity with *D. immitis* GST. The ORF of WbGST cloned in pRSET A was expressed as histidine tagged fusion protein. The recombinant fusion protein with the histidine tag had a molecular mass of approximately 28 kDa. SDS-PAGE analysis showed that expressed rWbGST consisted >20% of the total *E. coli* proteins. Subsequently, the recombinant protein was purified using metal affinity column chromatography to near homogeneity (Figure 2). After preparing purified rWbGST, GST enzyme activity was determined using CDNB and GSH substrates. Our results showed that purified rWbGST had significant enzyme activity (1.020±0.059 µM min^−1^ mg^−1^ of purified rWbGST protein). EST database search of *W. bancrofti* showed the presence of only phi- class of GST and did not show the existence of any other classes of GST. Therefore, based on the sequence data and enzymatic activity we believe that the GST cloned in this study belongs to Phi-class of GST.

### Humoral immune responses to rWbGST in endemic population and in immunized mice/jird sera

**Figure 3:**
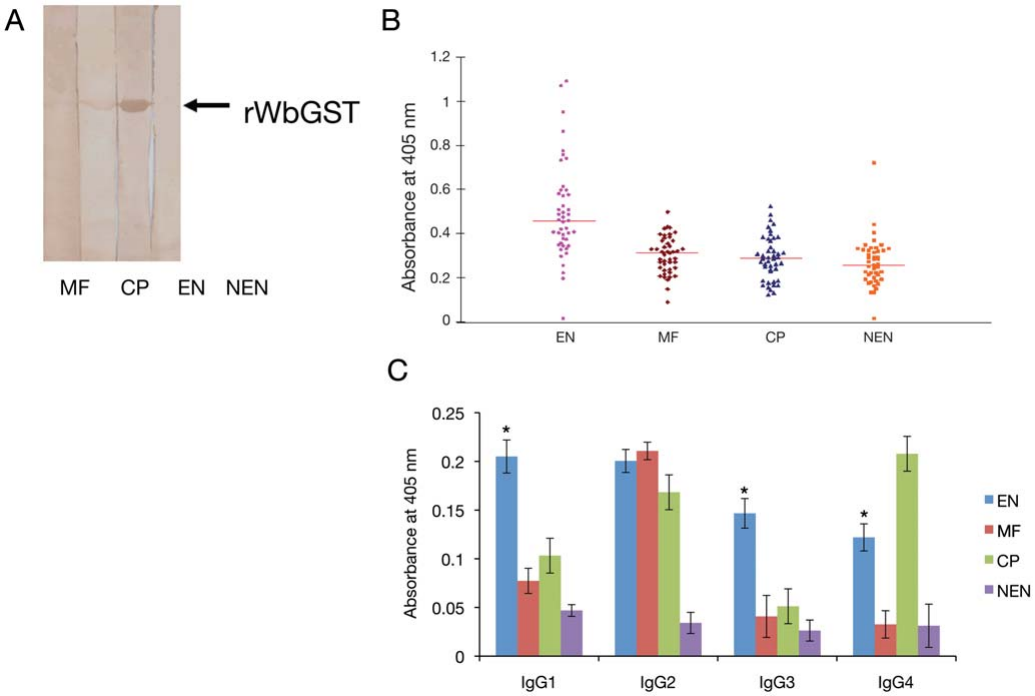
Human immune responses to WbGST. A. Immunoreactivity of rWbGST with sera from different clinical groups of bancroftian filariasis. Approximately 1 µg of rWbGST was resolved on 15% SDS-PAGE, transferred to nitrocellulose membrane and blots were probed with pooled sera from MF, CP, EN or NEN individuals. Results showed strong immunoreactivity with pooled EN sera followed by CP and MF but no reactivity was detected with control NEN sera. Data is representative of one of three experiments using the same sera samples. B. Humoral immune response to rWbGST in human subjects. Total IgG levels against rWbGST in various clinical groups were evaluated by ELISA. Each data point represents single individual absorbance from the four different groups. Horizontal lines represent geometric mean value of EN (43), Mf (45), CP (45) and NEN (39) samples respectively. C. WbGST-specific IgG subclasses in the sera from different clinical groups. Isotype of anti-WbGST IgG antibodies in the sera from various groups of human subjects (EN, MF, CP, TPE and NEN) were evaluated using an isotype-specific ELISA. Data presented is mean±SD value from EN (43), Mf (45), CP (45) and NEN (39). * Significant (p<0.005) compared to all the other three groups (CP, MF and NEN). The statistical significance was calculated by Kruskal–Wallis test.

Humoral immune responses to rWbGST were determined by western blot analysis and ELISA. Immunoblot analysis confirmed the presence of high levels of anti-WbGST antibodies in pooled EN sera. However, anti-WbGST antibodies were absent in pooled MF and NEN sera (Figure 3A). Sero-reactivity of individual samples was then evaluated by an ELISA. EN sera had significant amounts of anti-WbGST antibodies (p<0.001) (Figure 3B). Analysis of the isotype of IgG antibodies showed that the anti-WbGST IgG antibodies in the sera of EN individuals were predominantly of IgG1 and IgG3 isotype (Figure 3C), whereas, the IgG response from Mf individuals were predominantly IgG2 isotype. Interestingly, both EN and CP individuals had significantly elevated levels of anti-WbGST IgG4 antibodies in their sera.

**Figure 4:**
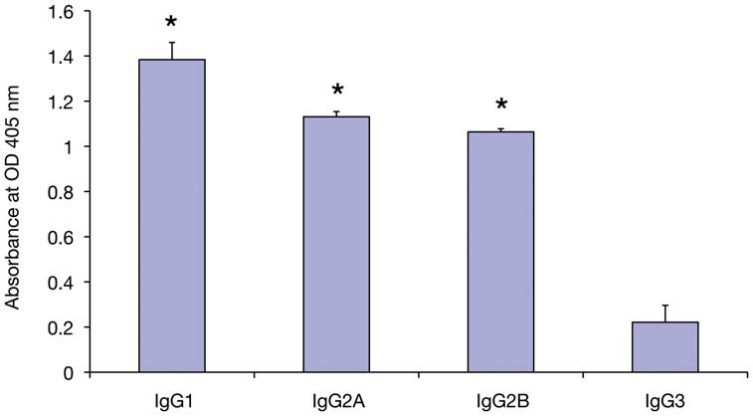
Subclass specific anti-WbGST IgG antibodies in the sera of mice immunized with rWbGST protein. Subclass analysis was performed using a mouse antibody isotyping ELISA kit. The bars represent the mean O.D.±SD at 405 nm of five mice per group.

Levels of anti-WbGST antibodies were also determined in the sera of mice and jirds by ELISA. Results showed that both mice and jirds immunized with rWbGST developed high antibody titer (1∶10000), whereas, control mice or jirds had no detectable WbGST specific antibodies in their sera. Isotype profile of anti-WbGST IgG antibodies showed that higher levels of both IgG1 (Th2) and IgG2a (Th1) anti-WbGST antibodies are present in immunized animals (Figure 4).

### Stage-specific expression of GST

**Figure 5:**
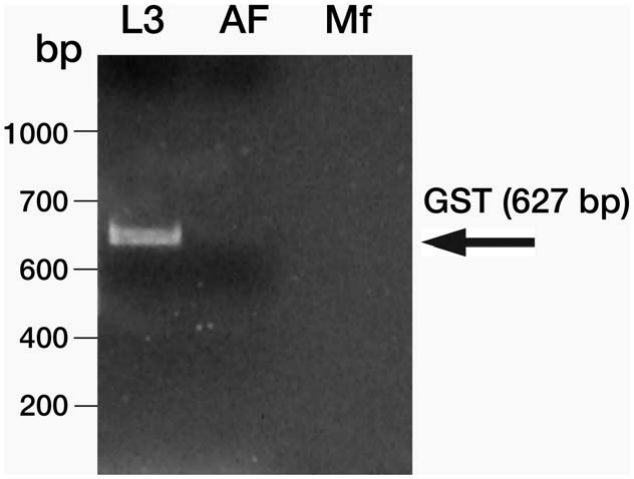
Stage-specific expression of filarial *GST*. *WbGST* gene was PCR amplified form *W.bancrofti* stage-specific cDNA libraries using WbGST gene specific forward and reverse primers. Results show that the *WbGST gene* is predominantly expressed in the L3 stage of the *W.bancrofti* and is barely detectable in adult female and microfilarial stages. Lane 1: 100 bp ladder; 2: *W. bancrofti* L3; 3: Adult Female; and 4: Mf.

Stage-specific expression of *GST* gene was investigated by PCR using gene-specific forward and reverse primers from the cDNA libraries of the various life stages of the *W. bancrofti*. This analysis showed that transcripts of the GST gene were present predominantly in the L3 stage of the parasite (Figure 5).

### Inhibition of GST enzymatic activity by anti WbGST antibodies

EN individuals carry antibodies against WbGST. To determine whether these antibodies are functional, we performed a neutralization assay. Results from these studies showed that sera from EN individuals and from mice immunized with rWbGST significantly inhibited WbGST enzyme activity compared to that by NEN individuals and sera from control mice (p<0.001). The level of inhibition of the GST enzyme activity by EN sera or immune mouse sera was only approximately 50% of NEN or pre-immune sera respectively.

**Figure 6:**
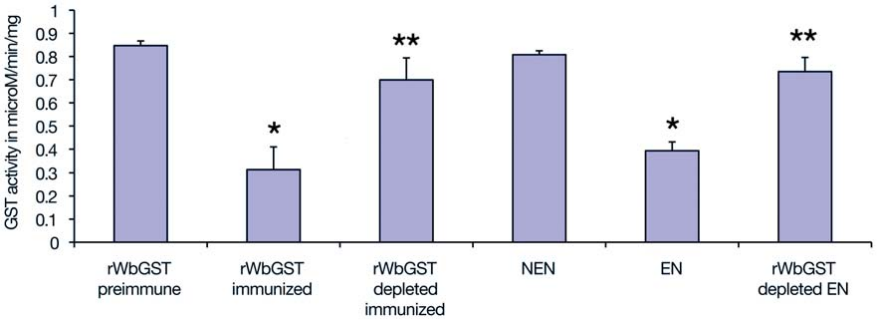
Anti-WbGST antibodies can neutralize the enzymatic activity of rWbGST. rWbGST was added to various test sera (pooled EN sera, sera from mouse immunized with rWbGST, EN and mouse sera depleted of anti WbGST antibodies) and incubated for 1 hr at 37°C followed by another incubation at 4°C for 4 h. Following incubation, GST enzyme activity was determined spectrophotometrically at 340 nm by measuring enzymatic conjugation of glutathione with 1-chloro-2, 4-dinitrobenzene (CDNB). Individual bar represents mean±SD of GST activity (µM/min/mg). Samples were read in triplicate wells. Data is representative of one of three similar experiments. Pre-immune sera from mice and sera from NEN individuals were used as control samples. * Significant (p<0.005) compared to pre-immune or NEN sera samples. ** significant (p<0.01) compared to non-depleted immune sera or EN sera samples.

To determine whether the GST inhibitory activity in the sera of EN individuals and immune sera was due to anti-WbGST antibodies, we first depleted WbGST specific antibodies from the sera of immunized mice and from pooled sera of EN individuals using rWbGST protein coupled to IMAC column. An ELISA analysis confirmed that all the anti-WbGST antibodies were completely depleted from the sera samples (data not shown). Depletion of anti-WbGST antibodies from the sera samples were also confirmed by western blot analysis (data not shown). The antibody depleted sera were then used in the GST enzymatic assay. These studies showed that depletion of anti-WbGST antibodies from EN sera and from sera of immunized mice resulted in the removal of GST enzyme inhibitory activity from these sera samples. The anti-WbGST antibody depleted sera behaved just like the NEN or pre-immune sera suggesting that the anti-WbGST antibodies were potentially responsible for the GST enzyme neutralizing activity (Figure 6).

### *In vitro* ADCC

**Figure 7:**
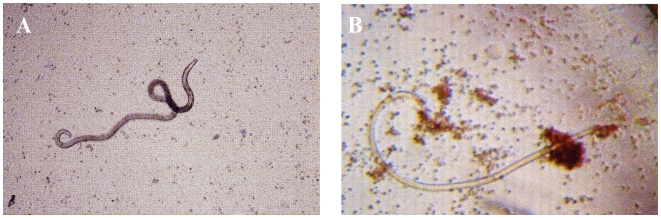
Photomicrograph of L3 recovered from cultures after ADCC assay. A. L3s incubated in NEN sera and PBMCs. There are no cells adhered to the larva and the larva was active. B. L3s incubated in EN sera and PBMCs. Note the cluster of cells adhered throughout the surface of larvae, but more specifically to the anterior and posterior end of the larva.

Previous studies showed that protective antibodies can kill filarial parasites by ADCC mediated mechanism [5],[25]. Since GST appears to be an important enzyme used by the parasite to neutralize the toxic molecules produced by activated cells in the host [26], we wanted to evaluate whether targeting the parasite GST with anti-WbGST antibodies can be lethal for the parasite or not. Thus in this study, we evaluated the heterologous cytotoxic effects of anti-rWbGST antibodies on *B. malayi* L3 using an *in vitro* ADCC assay. Results from these studies showed that sera from mice immunized with rWbGST promoted adherence of PEC to L3 larvae (Figure 7), and induced significant death of L3s (70.56% cytotoxicity) compared to L3s incubated with control sera (12.9%), (p<0.001). Depletion of anti-WbGST antibodies from the immune sera significantly reduced the L3 killing effect (21.43%), (Table 1). We also neutralized the activity of BmGST in the parasite by incubating *B. malayi* L3s with cDNB, a glutathione depleting agent and repeated the ADCC assay using immune mice sera and peritoneal cells. Results from these studies showed that neutralizing the effect of BmGST activity in the parasite with cDNB had no significant inhibitory effect on the ADCC ability of immune sera (Table 1). Similar results were observed when anti-WbGST-antibody-depleted EN sera were used in the ADCC assay. Pooled EN sera promoted adherence of PBMC's to L3 and induced 89.94% death of L3s. Depletion of anti-WbGST from the EN sera resulted in reduced L3 larval death (27.12%), (Table 2). In these studies also we found that neutralizing the activity of BmGST in the parasite by incubating *B. malayi* L3s with cDNB, had no significant inhibitory effect on the ADCC ability of EN sera (Table 2). These findings thus suggest that binding of specific anti-WbGST antibodies to promote cell mediated cytotoxicity is critical for the WbGST vaccine-induced protection against lymphatic filariasis infections.

**Table 1:** Results of antibody-dependent cellular cytotoxicity (ADCC) assay against heterologous *B. malayi* L3.

Groups (Pooled sera from 5 animals)	Live	Dead	Total	% Cytotoxicity (Mean±SD)
cDNB treated L3+rWbGST immunized mice sera+PEC	3	8	11	
	3	5	8	
	5	7	12	
				64.52±7.4*
L3+rWbGST immunized mice sera+PEC	2	6	8	
	3	7	10	
	3	6	9	
				70.56±4.19*
L3+rWbGST depleted mice sera+PEC	6	2	8	
	6	1	7	
	9	3	12	
				21.43±6.19**
L3+Alum Control sera+PEC	7	1	8	
	8	1	9	
	11	2	13	
				12.9±2.17

In these assays, L3s were incubated with pooled immune mouse sera and 1×10^5^ peritoneal cells. One group of L3s were treated with 14 µM of 1-chloro-2, 4-dinitrobenzene for 2 hrs then used in the ADCC assay.

*: Statistically significant (p<0.001) compared to alum controls.

**: Statistically significant (p<0.001) compared to non-depleted sera.

**Table 2:** Results of antibody-dependent cellular cytotoxicity (ADCC) assay against heterologous *B. malayi* L3.

Groups (Pooled sera of 10 individuals)	Live	Dead	Total	% Cytotoxicity (Mean±SD)
cDNB treated L3+EN sera+cells	3	7	10	
	2	9	11	
	3	6	9	
				72.82±7.96*
L3+EN sera+cells	1	9	10	
	1	12	13	
	1	7	8	
				89.94±2.40*
L3+rWbGST depleted EN sera+cells	9	3	12	
	8	2	10	
	7	4	11	
				27.12±8.39**
L3+NEN sera+cells	8	0	8	
	9	0	9	
	10	0	10	
				0

In these assays, L3s were incubated with pooled sera from EN individuals and 1×10^5^ PBMCs. One group of L3s were treated with 14 µM of 1-chloro-2, 4-dinitrobenzene for 2 hrs then used in the ADCC assay.

*: Statistically significant (p<0.001) compared to alum controls.

**: Statistically significant (p<0.001) compared to non-depleted sera.

### Adult worm recovery in immunized jirds

To evaluate the significance of the *in vitro* killing of L3s by anti-WbGST sera, we performed a vaccination trial and challenge experiment in jirds. Ten days after the last dose of immunization, jirds immunized with rWbGST or alum control were challenged with 100 L3 larvae. On day 120, infected animals were autopsied to determine the worm recovery. Mean adult worm recovery in rWbGST immunized jirds was only 6.17±0.42 whereas, control jirds had 16.0 ± 1.24 worms established (Table 3). This difference was statistically significant and accounted for about 61.44% reduction in parasite establishment in jirds immunized with rWbGST compared to those in control jirds (p<0.005).

**Table 3:** Data shows the number of adult worms recovered from jirds immunized with rWbGST or from control group of jirds after a heterologous challenge infection with *B. malayi* L3 larvae.

Jird groups	Adult worms recovered		Total Recovered	Mean worm burden	Percentage Reduction
Control	Male	Female			
Jird 1	8	5	13	16.0±1.24	
Jird 2	6	10	16		
Jird 3	11	8	19		
**Vaccinated**					
Jird 1	3	6	9	6.17±0.42	**61.44** *
Jird 2	2	5	7		
Jird 3	1	3	4		
Jird 4	2	4	6		
Jird 5	2	5	7		
Jird 6	1	3	4		

*: Significant difference (*p*<0.005, Mann–Whitney *U*-test).

## Discussion

Glutathione-S-transferases are extensively investigated as vaccine candidates against several parasitic infections [10]–[13]. The present study describes cloning of *WbGST* gene from the cDNA library of *W. bancrofti* and evaluating its potential as a vaccine candidate in experimental animal models and as well in human using sera samples from putatively immune individuals. Results from our studies show that immunization with rWbGST confers significant protection against a challenge infection with *B.malayi* in jird model. Similarly, we show that naturally occurring anti-WbGST antibodies in EN individuals can kill L3s of *B. malayi in vitro* through an ADCC mechanism.

*B. malayi* and *W. bancrofti* are closely related parasites and show significant antigenic overlap. Results of a blast search analysis showed that *WbGST* gene has 98% homology with *BmGST*. Similarly, antibodies generated against *W. bancrofti* antigens in infected and immune individuals show significant cross reactivity with *B. malayi* antigens [25]. Furthermore, since *W. bancrofti* infections are more prevalent than *B. malayi* infections in several parts of the world, we decided to test the prophylactic potential of rWbGST. It is practically impossible to maintain *W. bancrofti* life cycle in the laboratory, therefore, in these studies we used *B. malayi* for heterologous challenge infections.

PCR screening of the cDNA libraries of different life cycle stages of *W. bancrofti* showed that *WbGST* gene is expressed only in the infective L3 stages. Since targeting the infective stages can block the infection, WbGST appeared to be an attractive candidate for evaluating the vaccine potential. Before evaluating the vaccine potential we first determined whether WbGST retained the GST enzymatic activity. This is critical because parasite GSTs behave like mammalian GSTs in neutralizing oxidative radicals. By neutralizing these noxious substances, the parasites are believed to counteract the host mediated oxidative stress. Our results show that rWbGST has significant GST enzyme activity. These findings are similar to those reported for the trematode GSTs of *S. mansoni*, *S. japonicum* [27] and *F. hepatica* [28]. Interestingly, the specific GST activity (1.02 µmoles/min/mg) appears to be about 5–20% less than that reported for Schistosoma or Fasciola GSTs using the same substrate. WbGST cloned in this study belongs to Phi class. Phi-class of Sm28GST, Phi-class of Sj26GST and Phi-class of Fasiola GST all have low substrate specificities and inhibitor low sensitivities [27],[28]. This might explain our findings that Phi-type of GSTs in general have a low specific GST activity. Previous studies using all these trematode GSTs suggest that parasite GST is a potential vaccine candidate. Results from our current studies support these findings and show that GST from filarial parasites also has similar vaccine potential.

Putatively immune individuals (EN) carry antibodies to WbGST in their sera. Majority of the antibodies are either of IgG1 or IgG3 isotype suggesting that cytophilic type of antibodies may have a predominant role in the protection against this infection in human. These individuals also showed significant increases in anti-WbGST IgG4 antibodies similar to CP individuals. In another study also we observed a similar Th2 biased responses following immunization of mice with OvGST-2 of *O. volvulus* [29] or rBmALT of *B. malayi*. In these studies also EN individuals were the only one showing predominantly IgG1 and IgG3 antibody responses [30],[31]. Another interesting observation in the present study was that MF individuals carry IgG2 antibodies against WbGST in their sera suggesting largely a Th1 type response in these individuals compared to putatively resistant EN individuals who show a Th2 biased antibody responses in their sera.

Our studies also show that anti-WbGST antibodies in the sera of EN individuals and sera of immunized mice have the capability to neutralize parasite GST function. However, we failed to observe 100% inhibition of GST enzyme activity either with the EN sera or with the immune mice sera. This could be possibly because (1) the antibody titer was not optimized for maximum neutralization of GST activity or (2) possibly the anti-WbGST antibodies could only neutralize approximately 50% of the rWbGST activity. Nevertheless studies by Morrison *et al* [10] suggest that there is no correlation between titer of GST neutralizing antibodies and protection against *F. gigantica* infections in mice. Yet studies by Rathaur *et al* [29] showed that *S. cervi* GST activity could be inhibited by sera from jirds that are vaccinated with *S. cervi* GST. Similarly, Bal and Das [32] have also demonstrated inhibition of *S.digitata* GST activity when incubated with sera from individuals infected with *W. bancrofti*. Anti-*S. mansoni* 28 GST IgA antibodies can neutralize the parasite GST activity in human schistosomiasis and can impair *in vitro* hatching of *S. mansoni* eggs [17]. Thus, there appears to be some indication that neutralizing the GST activity may have adverse effect on the parasite. To test if neutralizing WbGST activity will interfere with protection, we performed an *in vitro* ADCC assay. Although this is not a direct measure of protection, it does provide some important information about the potential capabilities of anti-WbGST antibodies. Our *in vitro* results clearly show that human anti-WbGST antibodies can participate in the killing of infective L3s of *B. malayi*. Major confirmation of this finding comes from the fact that depletion of anti-WbGST antibodies from the EN sera eliminated the L3 killing effect. Since eliminating L3s are important in preventing the infection, this finding also suggests that anti-WbGST may have a crucial role in protection against this infection in EN individuals. In addition, we also neutralized the activity of BmGST in the parasite by incubating *B. malayi* L3s with cDNB, a glutathione depleting agent and repeated the ADCC assay using EN sera and PBMCs. Results from these studies show that neutralizing the effect of BmGST in the parasite with cDNB had no inhibitory effect on the ADCC ability of EN sera. Although in this study we did not test whether we neutralized all the parasite GST activity with cDNB, it appears that neutralization of GST activity is not critical for ADCC function. These findings were similar to that reported earlier by Morrison *et al* [10]. Thus, binding of specific anti-WbGST antibodies to the parasite that promote cell mediated cytotoxicity are critical for the WbGST vaccine-induced protection against lymphatic filariasis infections.

We were able to replicate the ADCC finding using a mouse model as well. The mouse anti-WbGST antibodies were also able to participate in the killing of *B. malayi* L3s *in vitro* and depletion of the anti-WbGST antibodies from the mouse sera removed the L3 killing effect from the immune sera. These findings further suggest that GST may be a critical protein for the survival of the *B. malayi* L3. In our studies we did not identify the effector cells. Given that majority of the anti-WbGST antibodies are of IgG subclass, further studies evaluating the Fcγ bearing cells might help identify the effector cells that participate in the ADCC mechanism.

Mouse is not a permissive host in that the worms do not mature into adults. Therefore, we did not conduct any challenge experiments in the mouse model. Instead, we used a jird model where the parasite matures into adult male and female parasites [33]. Vaccine studies in jirds showed that 61% protection could be achieved following immunization with rWbGST. Similar results have been reported following vaccination with candidate GST vaccine against other parasites [12],[16]. These findings thus suggest that WbGST may be a potential vaccine candidate. Our results support and are in agreement with previous vaccination trials using fasciola GST [10], schistosome GST [21],[27], and *O. volvulus* GST [29] that parasite GSTs are potential vaccine candidates for controlling certain helminth infections in animals and human.

In conclusion, results presented in this study show that putatively immune individuals in an endemic area carry antibodies against WbGST. These antibodies can neutralize the parasite GST activity and kill infective larvae of *B. malayi* that express the GST. Animal studies confirm that immunization with rWbGST can confer protection against a challenge infection. These findings thus suggest WbGST could be potentially developed as a vaccine candidate against lymphatic filarial parasites. Since only L3 stages appear to express the WbGST protein it is likely that this vaccination will be more effective in non-infected individuals as a preventive vaccine.
